# Supplementary material for: Robust Genetic Analysis of the X-Linked Anophthalmic (Ie) Mouse
Source: Genes (Basel). 2022 Oct 5;13(10):1797. doi: 10.3390/genes13101797 (PMC9601528; doi:10.3390/genes13101797)
Supplement: Supplementary file 1 [file genes-13-01797-s001.zip › genes-1878425-supplementary.pdf]

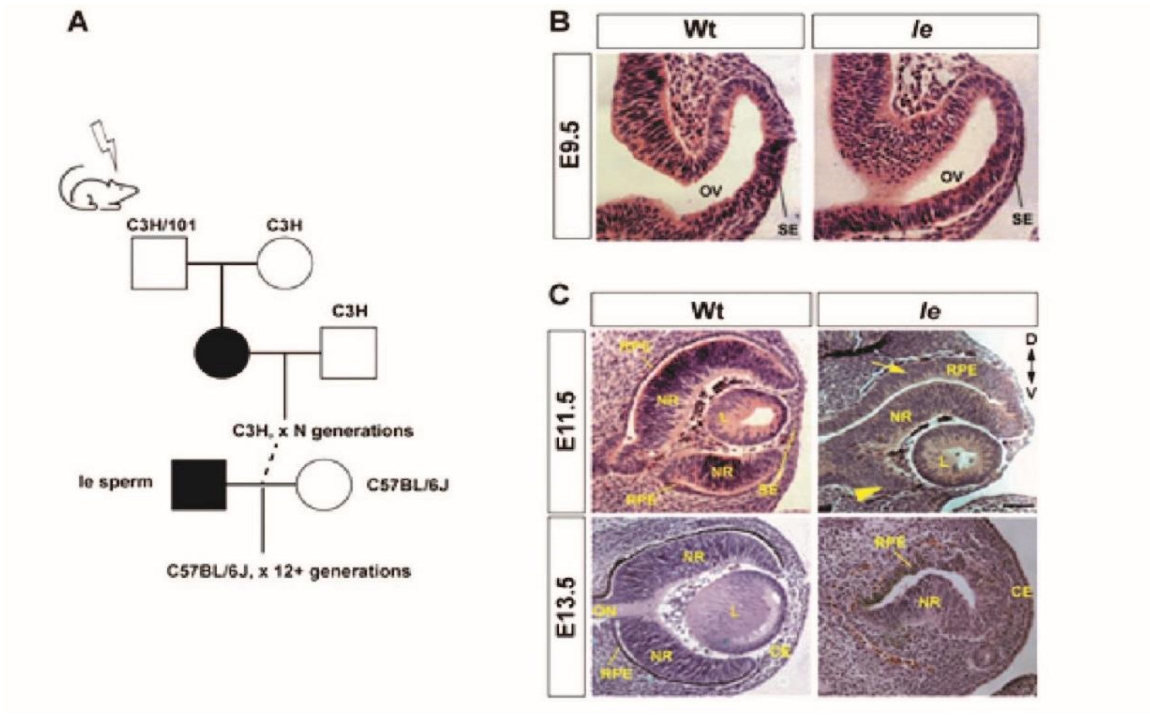

Supplemental Figure S1. ***Ie*** **mouse breeding and histology phenotype.** (**A**) Breeding schema for Ie. (**B**) Early eye development analysis by histology at E9.5 optic vesicle stage revealed no obvious differences between *Ie* and stage-matched wild type littermate control eyes. (**C**) Subsequent H&E analysis revealed hypoplasia of the ventral eye (arrowhead) and thickened RPE layer in the dorsal eye (arrow) at optic cup stage (E11.5) in *Ie*, and by E13.5 the *Ie* eye was severely malformed compared to wild type controls, with no normal eye structure and only residual RPE tissue in the dorsal region and abnormal neural retina in the ventral region. The corneal epithelia and overlying ectoderm appeared normal.

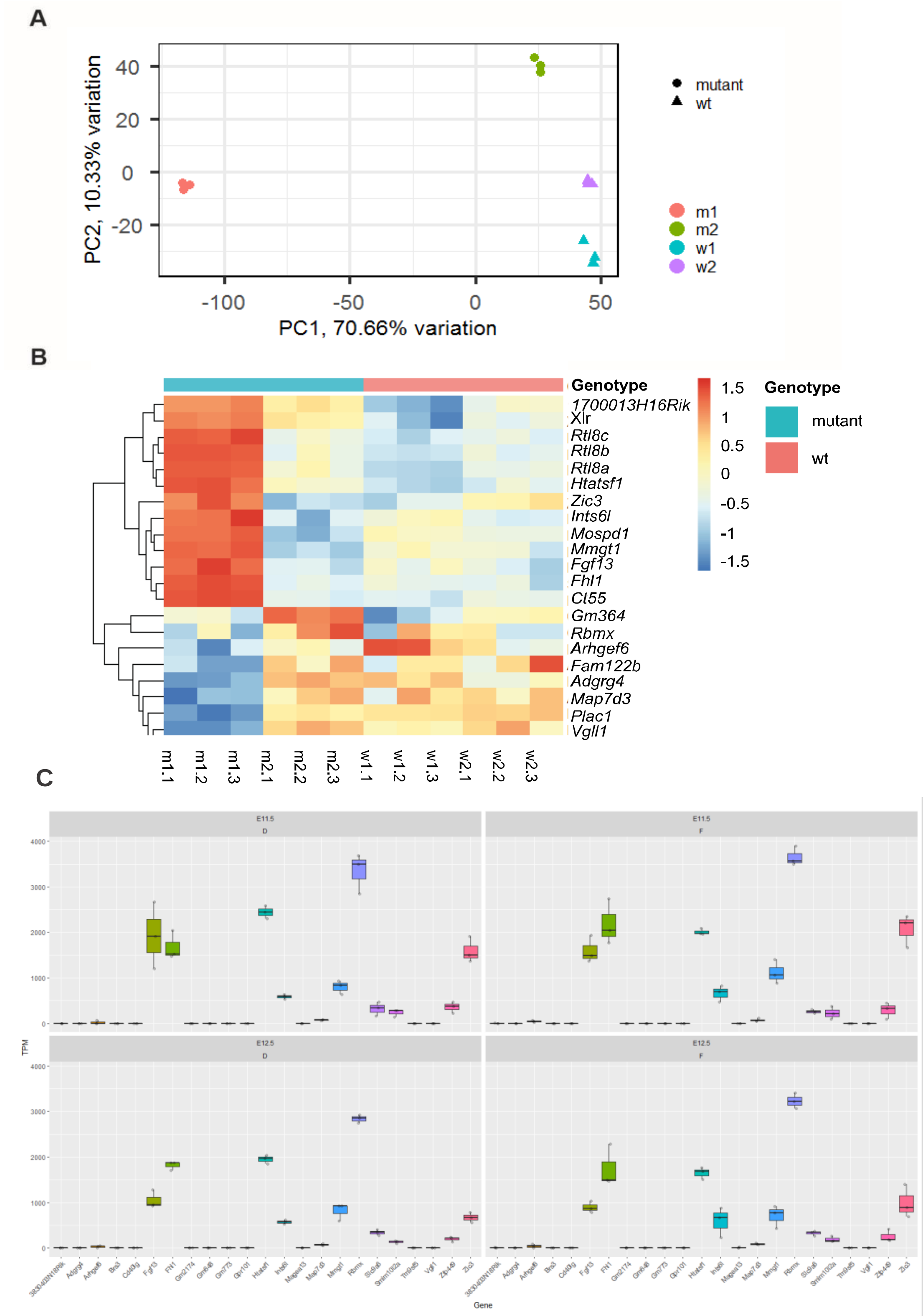

Supplemental Figure S2. Variability between replicates in *Ie* OV organoids. (**A**) Principal Component Analysis plot shows the variability between mutant (m1 and m2) and wild type (w1 and w2) *Ie* organoids biological replicates in RNA-seq data. (**B**) Heat map of gene expression within chrX: 56,145,000-58,385,000 critical interval between mutant and wild type OV organoids. (**C**) Expression analysis from mouse optic fissure (**F**) and dorsal optic cup (**D**) for genes located in the broader critical interval, taken from existing transcriptome data for wild type mice at embryonic stages E11.5 and E12.5.

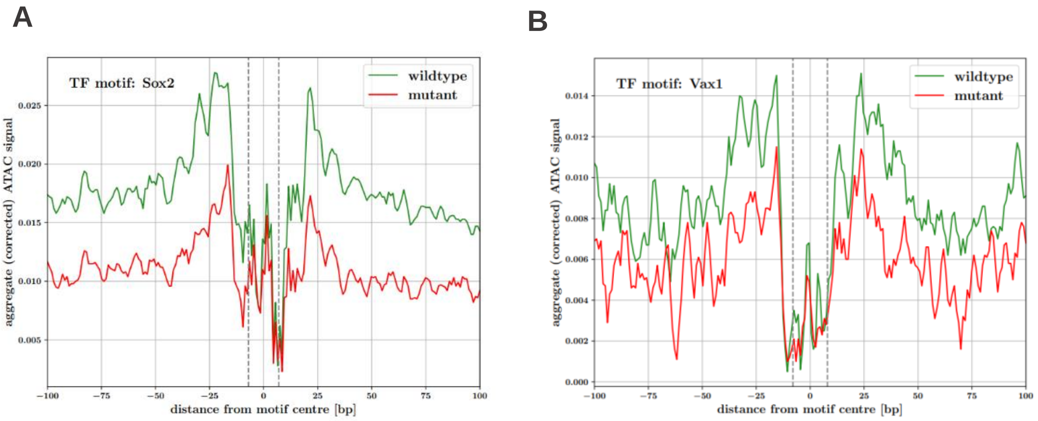

Supplemental Figure S3. **Sox2 motif footprint in wild type and mutant samples.** (**A**) Aggregate Tn5-bias corrected ATAC-seq signal around detected Sox2 motif occurrences in consensus peak regions for wild type (green line) and mutant (red line) samples. Vertical dashed lines indicate edges of the Sox2 motif. (**B**) Same as (**A**) for Vax 1 motif occurrences. Vertical dashed lines indicate edges of the Sox2 (**A**) and Vax1 (**B**) motifs.

Table S1. SVs call in chrX critical interval.

Position in ChrX (GRCm38)	Variant type	SV length (bp)	Gene	Predicted consequence
**53704314**	Insertion[1]	1842	*AW822252*	intron_variant
**53963871**	insertion	33	*Gm14591*	downstream_gene_variant
**54096640**	Duplication[1]	9772	*Gm14644*	downstream_gene_variant
**57101887**	deletion	39	*Vgll1*	feature_truncating
**57259853**	deletion	38	*Arhgef6*	intron_variant
**57535040**	insertion	54	--	intergenic variant
**57742997**	Insertion[2]	459	*Gm8111*	downstream_gene_variant
**57790180**	insertion	34	--	intergenic_variant
**57824566**	Insertion[2]	107	--	intergenic_variant
**57836683**	insertion	136	--	intergenic_variant
**57855212**	Insertion[2]	428	--	intergenic_variant
**57940202**	insertion	34	--	Intergenic_variant
**58043500**	insertion	48	*Zic3*	downstream_gene_variant
**58043875**	insertion	34	*Zic3*	downstream_gene_variant
**58109931**	deletion	38	--	intergenic_variant
**58137629**	insertion	36	--	intergenic_variant
**58178827**	deletion	40	--	intergenic_variant
**58923847**	insertion	33	*4930550L24Rik*	downstream_gene_variant
**58966466**	deletion	34	--	intergenic_variant
**58967642**	insertion	6464	--	intergenic_variant
**59054939**	insertion	34	--	Intergenic_variant
**59168195**	deletion	49	*Fgf13*	intron_variant

[1]Similar SV have been reported on this locus in the mouse genomes project https://www.sanger.ac.uk/sanger/Mouse_SnpViewer/rel-1505.

[2]SV reported in the mouse genomes project.

Table S2. Indels found in autosomal and chrX critical region in *Ie* mouse wild type and mutant.

***Ie* status**	**Region (GRCm38)**	**indels**	**ins:del ratio**	**MUT:WT**
**Wild type**	autosomes	7608	1.7	3.7
	chrX	118	1.5	24.9
	X:57000000 -60000000	0	-	∞
**Mutant**	autosomes	28405	1.5	
	chrX	2934	1.2	
	X:57000000 -60000000	221	1.2	
